# “When I do have some time, rather than spend it polishing silver, I want to spend it with my grandkids”: a qualitative exploration of patient values following left ventricular assist device implantation

**DOI:** 10.1186/s12904-024-01454-y

**Published:** 2024-05-22

**Authors:** Avery C. Bechthold, Colleen K. McIlvennan, Daniel D. Matlock, Deborah B. Ejem, Rachel D. Wells, Jesse LeJeune, Marie A. Bakitas, J. Nicholas Odom

**Affiliations:** 1 School of Nursing, University of Alabama at Birmingham, 1720 2nd Avenue South NB 350, Birmingham, AL 35294 USA; 2 Division of Cardiology, University of Colorado Anschutz Medical Campus, Aurora, CO USA; 3 Adult and Child Consortium for Health Outcomes Research and Delivery Science, University of Colorado Anschutz Medical Campus, Aurora, CO USA; 4 Division of Geriatric Medicine, University of Colorado School of Medicine, Aurora, CO USA; 5 Cardiology Clinic, UAB Hospital, Birmingham, AL USA

**Keywords:** Beliefs, Communication, Goals, Shared decision-making, Ventricular assist devices, Patient-centered care, Qualitative research, Self-care, values

## Abstract

**Background:**

Values are broadly understood to have implications for how individuals make decisions and cope with serious illness stressors, yet it remains uncertain how patients and their family and friend caregivers discuss, reflect upon, and act on their values in the post-left ventricular assist device (LVAD) implantation context. This study aimed to explore the values elicitation experiences of patients with an LVAD in the post-implantation period.

**Methods:**

Qualitative descriptive study of LVAD recipients. Socio-demographics and patient resource use were analyzed using descriptive statistics and semi-structured interview data using thematic analysis. Adult (> 18 years) patients with an LVAD receiving care at an outpatient clinic in the Southeastern United States.

**Results:**

Interviewed patients (*n* = 27) were 30–76 years, 59% male, 67% non-Hispanic Black, 70% married/living with a partner, and 70% urban-dwelling. Three broad themes of patient values elicitation experiences emerged: 1) LVAD implantation prompts deep reflection about life and what is important, 2) patient values are communicated in various circumstances to convey personal goals and priorities to caregivers and clinicians, and 3) patients leverage their values for strength and guidance in navigating life post-LVAD implantation. LVAD implantation was an impactful experience often leading to reevaluation of patients’ values; these values became instrumental to making health decisions and coping with stressors during the post-LVAD implantation period. Patient values arose within broad, informal exchanges and focused, decision-making conversations with their caregiver and the healthcare team.

**Conclusions:**

Clinicians should consider assessing the values of patients post-implantation to facilitate shared understanding of their goals/priorities and identify potential changes in their coping.

**Supplementary Information:**

The online version contains supplementary material available at 10.1186/s12904-024-01454-y.

## Background

In 2017, over 16,000 individuals worldwide were registered as having a left ventricular assist device (LVAD), a surgically implanted pump supporting heart function and blood flow [1, 2]. Patients with advanced-stage heart failure who are unresponsive to drug therapies, ineligible or waiting for cardiac transplantation, and without certain contraindications, may be considered for an LVAD. Despite improvements in survival and device malfunction among newer LVAD devices, patients and their family/friend caregivers face significant lifestyle changes and self-care decisions post-implantation, including routine daily tasks, such as checking the device and changing dressings to monitoring for serious complications such as hemolysis, pump thrombosis, infection, and gastrointestinal bleeding [2–4]. This transition can be particularly challenging for LVAD recipients who are African American and/or rural-dwelling as they have higher rates of discharge to skilled nursing facilities or home health care compared to white individuals; and have more frequent emergency department visits compared to urban-dwellers [5–7].

Palliative care has been recommended by ISHLT guidelines to support patients and caregivers in adjusting to life changes post-LVAD implantation and has been found to improve quality of life, symptom burden, mood, and spiritual well-being among LVAD recipients [8, 9]. However, specialist palliative care consults remain low both pre- (~ 4%) [8] and post-implant (~ 11%) [10], particularly among African American and Hispanic individuals. A key element of high-quality, patient-centered care—and palliative care—is proficiency in effectively eliciting and incorporating patient values, goals, and preferences into the care plan [11]. Values, defined as who/what matters most [12], are broadly understood to have implications for how individuals make health-related decisions and cope [4, 13, 14]. Values elicitation, an intentional process of exploring one’s core beliefs [15], helps identify care priorities and align care/treatment decisions with patients’ goals [16, 17]. Recognizing the importance of values in decision-making and coping processes, it is critical to understand how patients discuss, reflect upon, and act on their values post-LVAD implantation.

In this context, we conducted a qualitative descriptive study exploring the values elicitation experiences of patients with an LVAD in the post-implantation period. To our knowledge, this is the first study to explore patient values following LVAD implantation. Findings may provide palliative care clinicians insight into the values patient prioritize post-implantation and how these values impact their self-care and coping.

## Methods

Following qualitative reporting guidelines [18], this paper explores the experiences of LVAD recipients [19, 20]. The study protocol and data safety plan were approved by the University of Alabama at Birmingham Institutional Review Board (IRB-300,010,359). Participants gave written or verbal consent.

### Study design

Qualitative analysis of semi-structured interviews of how values are discussed, reflected upon, and acted on among patients who were LVAD recipients. A qualitative exploration facilitated a comprehensive understanding of patient values elicitation experiences given a lack of existing data on the topic.

### Setting and sample

Patients were recruited from an outpatient clinic in the Southeastern U.S. As one of few regional centers providing mechanical circulatory support, the clinic serves a diverse patient population.

### Recruitment

From January 2023 to July 2023, A.B. screened and approached potentially eligible patients in-person before or immediately after their clinic visit. Inclusion criteria are listed in Table 1. To ensure inclusion of diverse participants, we used purposive sampling [21] of all patients currently implanted with an LVAD. Based on similar studies [22, 23], we expected data saturation [24] after enrollment of 20–30 patients. Participants received $50 total for study completion, which aligns with other heart failure and LVAD studies [25, 26].

**Table 1:** Patient inclusion and exclusion criteria

Inclusion	Exclusion
1) Implanted with an LVAD; 2) ≥ 18 years; 3) English-speaking	1) Documentation in the electronic medical record or self-endorsing of substance use, active severe mental illness, dementia/confusion, suicidal ideation, severely impaired communication skills (e.g., aphasia), or uncorrected hearing loss; 2) Actively enrolled in hospice and/or a clinician-determined prognosis of less than 6 months

*LVAD *left ventricular assist device

### Data collection

Patients completed sociodemographic and resource use questions. Open-ended interview questions focused on LVAD management, personal values, values discussions with caregivers and healthcare teams, and impact of values on health decisions and coping. The interview guide (Additional file 1) was developed by our interdisciplinary team and based on an adapted conceptual model derived from the situation-specific theory of heart failure self-care [4] and other relevant literature [18, 27]. This model emphasizes the influence of patient-level factors (e.g., values) and environmental factors (e.g., family, healthcare team) on patient self-care behaviors [4].

The interview guide was revised according to emerging themes and participant feedback after each interview. For example, it was determined that the word ‘values’ should be replaced with specific values mentioned by participants, such as ‘family’, once elicited. Participants were also asked to share their reaction to the responses of other participants (e.g., “Some people I’ve interviewed have said that they have thought about their health more since they received their LVAD. What are your thoughts about this?”). Interview reflections were recorded after each interview (Additional file 1).

### Data analysis

#### Quantitative analysis

Descriptive statistics summarized sociodemographics and resource use. Continuous variables were presented as median ± standard deviation (SD), while categorical variables were presented as frequencies and percentages. Analyses were conducted using R (version 4.2.0) in RStudio.

#### Qualitative analysis

Transcribed verbatim interviews were reviewed for accuracy and analyzed using NVivo 12 and Attride-Stirling’s thematic analysis approach [28]. A deductive coding scheme was developed based on the following interview guide topics: values reflections, values discussions, and acting on values. The codebook helped reduce data into meaningful text segments (codes) which were reviewed by our multidisciplinary team for completeness and non-redundancy. Subsequent analyses consisted of 3 stages: (1) text reduction (coding materials, identifying themes, and constructing thematic networks), (2) text exploration (describing, exploring, and summarizing thematic networks), and (3) integration of exploration (interpreting patterns).

Thematic saturation was achieved once data created little or no change to the codebook and no new themes emerged [24]. Trustworthiness and authenticity were ensured using various strategies: [29] an audit trail documenting research decisions (Additional file 2), thick descriptions, and triangulation of findings among our interdisciplinary team. Member check forms were returned by 7 of 25 agreeable participants. Responses indicated support for the findings.

## Results

**Fig. 1:**
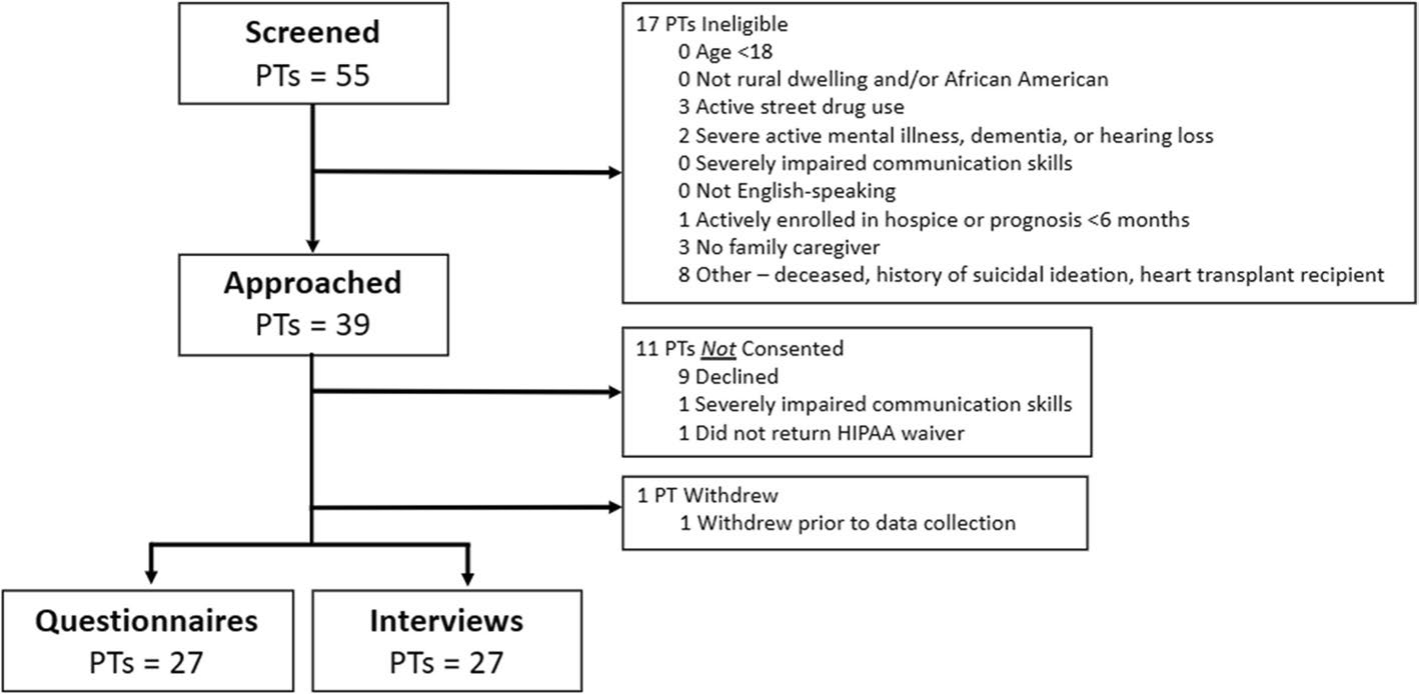
Participant screening, enrollment, and data collection. Note. HIPAA = Health Insurance Portability and Accountability Act; PT = patient

From January to July 2023, 39 patients were approached and 28 consented (Fig. 1). One patient withdrew during data collection, leaving 27 patients’ data evaluable. Patients were on average 49 years old, mostly male (*n* = 16; 59.3%), and non-Hispanic Black (*n* = 18; 66.7%). Additional characteristics are listed in Table 2. Interviews ranged 23 to 71 min (mean = 49 min). The overarching theme was that LVAD implantation was an impactful experience often leading to reevaluation of patients’ values; these values became instrumental to making health decisions and coping with stressors post-LVAD implantation. Three themes emerged during analysis (Table 3): 1) LVAD implantation prompts deep reflection about life and what is important, 2) patient values are communicated in various circumstances to convey personal goals and priorities to caregivers and clinicians, and 3) patients leverage their values for strength and guidance in navigating life post-LVAD implantation.

**Table 2:** Patient characteristics

Characteristic	No. of Patients (%), *n* = 27
Age: Median ± SD, y	49 ± 13.0
Sex, No. (%)	
Male	16 (59.3)
Female	11 (40.7)
Race, No. (%)	
Black	18 (66.7)
White	9 (33.3)
Hispanic/Latino, No. (%)	0 (0.0)
Rural-dwelling, No. (%)	8 (29.6)
Marital status	
Married/living with partner	19 (70.3)
Never married	1 (3.7)
Separated/divorced	6 (22.2)
Widowed	1 (3.7)
Education, No. (%)	
* ≤*High school	8 (29.6)
Some college	8 (29.6)
College or technical school	8 (29.6)
> College degree	3 (11.1)
Household income, No. (%)	
Under 29,999	13 (48.1)
30,000–49,999	1 (3.7)
50,000–74,999	7 (25.9)
75,000-150,000	5 (18.5)
Over 150,000	1 (3.7)
Employment, No. (%)	
Full-time	4 (14.8)
Part-time	1 (3.7)
Retired	8 (29.6)
Unemployed due to disability or illness	14 (51.9)
Insurance,^a^ No. (%)	
Medicare	20 (74.1)
Medicaid	9 (33.3)
Military	1 (3.7)
Private/commercial	4 (14.8)
Other	10 (37.0)
Religious affiliation, No. (%)	
Protestant	14 (51.8)
Christian, non-denominational	6 (22.2)
Pentecostal	2 (7.4)
Other	5 (18.5)
Cardiology clinic visits, past 3 months	
In-person, median (range)	2 (1–15)
Telephone, median (range)	1 (0–15)
Minutes from cardiology clinic, median (range)	105 (10–300)
Urgent care visits, past 3 months, median (range)	0 (0–1)
Emergency room, past 3 months, median (range)	0 (0–7)
Hospitalizations, past 3 months, median (range)	0 (0–40)
Intensive care unit, past 3 months, median (range)	0 (0–60)
Specialist palliative care, No. (%)	
Yes, currently/previously	9 (33.3)
No, never	14 (51.9)
Don’t know	4 (14.8)

Characteristics do not always add to 100% due to rounding

^a^Does not add to 100%, patients were able to select multiple items

**Table 3:** Basic, organizing, and global themes

Basic Themes	Organizing Themes	Global Themes
Values encompass the meaningful connections and experiences that give life significance	LVAD implantation evokes gratitude for each day spent with loved ones	LVAD implantation prompts deep reflection about life and what is important
Cherishing time with loved ones		
Embracing each day with gratitude		
Adopting a healthier lifestyle		
A shift towards prioritizing meaningful relationships	LVAD implantation prompts a (re)evaluation of what is most important in life	
Increased focus on personal well-being and happiness		
Increased empathy and awareness of others’ struggles		
Reaffirmation and reinforcement of lifelong core beliefs		
Values arise organically during day-to-day discussions	Patient values emerge organically during casual conversations and small talk	Patient values are communicated in various circumstances to convey personal goals and priorities to caregivers and clinicians
Values emerge during clinic visits in casual conversation and small talk		
Values conveyed to the care partner help rationalize a particular decision option	Patient values are conveyed in the context of health decisions	
Values discussions help convey personal goals and priorities to the healthcare team		
Values serve as guiding principles for navigating life and making choices	Patients pursue decision options aligned with their priorities	Patients leverage their values for strength and guidance in navigating life post LVAD-implantation
Making lifestyle adjustments to maximize health benefits and minimize risks		
Weighing health against other values and others’ values		
Avoiding stress to promote health and well-being		
Considering the impact on family		
Leveraging spiritual beliefs for strength and guidance	Patients draw strength and comfort from their spiritual beliefs Patients leverage meaningful relationships and activities to manage stress	
Finding comfort and strength through spiritual practices		
Receiving support and reaffirmation from care partner		
Leveraging relationships for support and comfort		
Family as a source of strength and driving force behind the will to live		
Engaging in enjoyable activities to manage stress		
Appreciation for life overshadowing difficulties		
Longing for restoration and normalcy	Inability to pursue what is important leads to feelings of frustration	
Pursuit of activities as a source of frustration		

### LVAD implantation prompts deep reflection about life and what is important

Patients expressed LVAD implantation had a profound impact on how they thought about life and their personal values. Relationships, health, and life were three values that arose as particularly meaningful. Patients endorsed increased awareness of the fragility of life and a shift or reaffirmation of their lifelong values. LVAD implantation emerged as a transformative life event with implications for patients’ values.

Patients were grateful for a second chance at life to enjoy additional time with loved ones and adopt a healthier lifestyle. Many shared a desire to experience as much as possible with the additional time afforded by their device. Recognizing that each day could be their last, patients expressed profound gratitude for each day:

‘*To wake up every morning and see a new day and learn to accept life and be grateful. It means so much to me now where I took it for granted before I got my LVAD. I didn’t think much about waking up. Every day now I wake up and I’m grateful and I’m thankful.*’ (PT28, 53-yr-old Black Female)

Two other values patients reported thinking about since receiving their LVAD were ‘health’ and ‘wellness’. Specifically, some patients were motivated to adopt a healthier lifestyle going forward. Many patients described an increased focus on making lifestyle changes to optimize their physical health and overall well-being:

‘*It’s changed a big way, because like I said before, I was being careless. I wasn’t taking good care of myself. That’s what led to the LVAD. Wasn’t taking care of yourself, just drinking the beer, wine, smoking. After that, I finally gave it all up since I had my LVAD, so that taught me a great lesson. If I wanna live, I have to sacrifice something.*’ (PT2, 69-yr-old Black Male)

While some patients reported certain values, such as ‘relationships’ and ‘health/well-being’, changed by becoming important, several patients reported a shift towards prioritizing relationships with loved ones and a higher power. Specifically, some described the value of ‘work’ becoming less important, whereas ‘family’ gained importance:

‘*There are people that I guess, live to work, and then there are those that work to live. I was definitely a live to work person before, and that’s not the case anymore. […] For the longest time my career was very, very important to me, and I would say now my family, and my own well-being is more important and it’s secondary.*’ (PT17, 49-yr-old Black Female)

Having gained an increased understanding of the struggles of others, either through their own illness journey or that of other patients, patients described a shift towards being more empathetic:

‘*Before, I didn’t really—maybe I took it a little lightly, takin’ that people were having surgeries and different ailments or whatever—disabilities or all these different things, I think [LVAD implantation] just made me a little bit more aware and conscious of their day-to-day struggles.*’ (PT20, 44-yr-old Black Male)

Finally, some patients endorsed not experiencing a shift in specific values, instead they reported their values either remained the same or all their values became more important. Several patients shared that LVAD implantation either reaffirmed or reinforced their lifelong values:

‘*I would not say that they have changed or been reprioritized. I say they’ve just been strengthened. They’ve just been strengthened. That’s the best way to put it.*’ (PT29, 43-yr-old White Male)

### Patient values are communicated in various circumstances to convey personal goals and priorities to caregivers and clinicians

Patients found values discussions offered a means to convey their personal goals and priorities in various contexts. Their values arose in both broad and focused contexts, including casual conversations with caregivers, small talk with their healthcare team, and in the context of specific health decisions.

Values often arose during broad, informal conversations. Many patients described their values emerging organically during casual, day-to-day discussions with their caregiver:

‘*To me it’s normal, it’s just normal conversation. We talk about life things all the time, and what we consider to be values, morals, ethics, honor […] It’s just part of our, like I said, day-to-day conversation.*’ (PT17, 49-yr-old Black Female)

Similarly, values discussions occurred in casual exchanges and small talk with the healthcare team. Oftentimes, patients endorsing such discussions described having a close relationship with the LVAD team, particularly the LVAD coordinators. During these conversations, patients and team members often exchanged updates about their families or hobbies:

‘*We talk about family, we talk about like sports, we talk about day to day. We do talk about the appointment of course, but there are other things, because they are an extended part of me, because I have to talk to them and see them so often, so I’m like, I just don’t want it to be business, business, business, business. I’m like, okay, well, how are you guys? Well, how’s your family? We just know so much about each other. Like I said, I’m really big on faith and family and friends, so they are in it right there, because they are an extended part of my family and my friends.*’ (PT8, 40-yr-old Black Female)

Patient values also arose during focused discussions. When faced with a health decision, such as LVAD transplant or heart transplant, patients shared their values with their caregiver to rationalize a particular decision option by conveying their perspective or stance:

‘*I told her back when we first got together I was going to do everything I could to make her happy—and when the doctor came in and told us, “This is it, your heart’s wore out,” […] I didn’t know what it was gonna cost, and I said, “It don’t matter, we’ll sell everything to pay for it because none of this matters if I’m not here to take care of you and the kids and everything.“*’ (PT25, 72-yr-old White Male)

Similarly, patients shared their values with the healthcare team to convey their goals and priorities—that is, who (e.g., family) or what (e.g., getting a heart transplant) is most important to them:

‘*The only thing I feel like I’ve really talked to my healthcare team about is my family. They allowed rules to be bent a little bit so that I could see my kids before and after surgeries. […] That was a wonderful thing, to say the least, because if I hadn’t woken up from the LVAD surgery, I would’ve at least gotten to say bye to my kids and they would’ve gotten to say bye to me.*’ (PT29, 43-yr-old White Male)

### Patients leverage their values for strength and guidance in navigating life post-LVAD implantation

Patient values acted as both strengthening and guiding forces in navigating life following LVAD implantation. Patients pursued decision options and coping strategies aligned with their values. While some became frustrated when pursuit of their values was not possible, others’ appreciation of life overshadowed the challenges and limitations that accompanied the LVAD.

Patients overwhelmingly discussed prioritizing decision options aligned with two values: ‘health’ and ‘family’. Patients adjusted their lifestyle to maximize their health and well-being and minimize potential health risks. For example, some limited their travel due to fear of health complications while away from their healthcare team. Others made lifestyle changes, such as giving up previously enjoyed hobbies (e.g., swimming, fishing) due to a risk of dislodging or damaging their device, or modified their diet:

‘*I think about the consequences I guess, as it relates to health. I know that there are certain things that I cannot have, that’s about part of having the LVAD. For example, like greens and salads, things that I really like, I have to eat that in very, very mild due to the vitamin K […] it makes me do what I’m supposed to do with respect to that.’* (PT17, 49-yr-old Black Female)

Decisions often involved weighing ‘health’ against other values, such as ‘pleasure’ (e.g., consuming unhealthy foods/beverages) or ‘work’. Adhering to dietary recommendations and avoiding overexertion were two particularly challenging restrictions. Avoidance of stress was another manifestation of patients acting on their ‘health’ value. Recognizing that stress is detrimental to one’s health/well-being, many patients engaged in enjoyable activities as an outlet or distraction from stress:

‘*I don’t have stress. I try to stay away from it. I’m just really relaxed. I get a book or something, get in front of the TV. I love sports. I really relax and just watching sports.*’ (PT2, 69-yr-old Black Male)

Family was described as impacting health decisions in two ways. First, many patients included family members when making health decisions, particularly the initial placement of the LVAD. Some patients made decisions together with their caregiver, whereas others received input from several family members. Oftentimes, patients considered the values of other individuals, typically their caregiver. Second, several patients considered the impact of decisions on their family. Specifically, patients strived to make decisions that promoted their own health/well-being, so they could stay alive for their family—especially their children:

‘*Ultimately, I wanna be here for my family […] I just had a baby in February, and she’s not even two months old yet. I’m like, “I gotta try to extend my life as long as possible to be a part of her life so that I can help mold this human bein’ I helped create.” I just feel like I gotta take the right steps to be a positive impact on their lives, as well as people in my family that’ve been impacts on my life.*’ (PT20, 44-yr-old Black Male)

For many patients, faith/spirituality played an integral role in how they coped with the stress of receiving their LVAD. Several patients shared they had thought a lot about their faith and relationship with a higher power. Spiritual practices, such as attending church, participating in services, and engaging in prayer, offered strength and comfort:

‘*I’ve been doing what the doctors tell me to do, but I’ve had many people all over—we have lots of friends scattered all over the southeast. Most of them go to church, and they’ve all had their churches pray. They’ve been praying for me. I attribute those prayers and God listening and granting those prayers to one of the major reasons why I was able to heal.*’ (PT29, 43-yr-old White Male)

Relationships was another key value leveraged for strength and guidance. Patients expressed a strong sense of support from their caregivers and felt particularly reassured when their values, underlying a particular decision option, aligned with those of their caregiver. In addition to caregiver support, patients often discussed leveraging meaningful relationships with family members, friends, and the LVAD team. Family was a particularly impactful source of strength. Patients often talked about their family—especially their children—as a driving force behind their will to live and persist in overcoming difficulties:

‘*I say it’s like the three Fs, my faith, my family, my friends. When we speak of values it’s like, I guess you can say like what I place on myself with those things, because those were the things that helped me through, and that continues to help me […] We joke about it now, but like my mom, she was saying how I would fight myself out of the anesthesia […] I wanted to fight for my life, because I have kids, and I’m like, I have to be here for them.* (PT8, 40-yr-old Black Female)

Besides relationships, patients pursued a range of enjoyable activities and hobbies when faced with frustrating or stressful situations (e.g., individual, group, indoor, outdoor). Activities were used as an escape or distraction from stress, or an outlet for managing stress. However, some patients expressed frustration at their inability to engage in activities since their LVAD was implanted, including a previously held job, household tasks, or hobbies. Oftentimes, activities were linked to a family role (e.g., breadwinner, homemaker) or a previous outlet for stress or way of connecting with others:

‘*I used to be an outdoorsman, and knowin’ that I can’t get on my kayak and go down a white water river, and go down there with my buddies, them buddies, a different type of buddies, it gets me upset, but I don’t think about it. I try not to anyways.*’ (PT7, 45-yr-old White Male)

Several patients expressed a longing for restored function and abilities. Often, these patients spoke about feeling restricted by the LVAD and wished for a heart transplant (or recovery), so they could engage in previously held family roles or hobbies. However, some patients discussed how their appreciation for life overshadowed the difficulties:

‘*Being alive and breathing with family members, it outweighs everything. Whether rich or poor, or sick or healthy, to be together, that’s very important.*’ (PT10, 42-yr-old Black Male)

## Discussion

### Main findings of the study

Our analysis of 27 interviews revealed that LVAD implantation was highly impactful and often prompted reevaluation of patient values. While some reported that their ‘relationship’, ‘health’, and ‘life’ values became more important, others found their pre-implantation values remained stable or all their values became more important. Values arose within broad, informal exchanges and focused, decision-making conversations with caregivers and the healthcare team to express personal goals and priorities. Patient values served as strengthening and guiding forces for navigating life post-LVAD implantation, although inability to pursue one’s values emerged as a source of frustration. These findings offer palliative care clinicians insight into the values patients might prioritize post-implantation and their potential impact on patient self-care and coping.

### What this study adds

LVAD implantation prompted contemplation about life and patient values. For many, the experience instilled a sense of gratitude for each day spent with loved ones, and provoked reevaluation of their priorities. This aligns with prior literature indicating significant life transitions (e.g., parenthood, migration) can lead to values shifts, which otherwise remain relatively stable [30, 31]. While less studied, serious illness can be highly disruptive and prompt reflections about ‘family’ and ‘quality of life’ values [32, 33].

Although some patients endorsed values shifts, others reported values stability post-implantation. Similar findings have been reported among patients considering LVAD with the majority reporting stability in their aggressiveness of care values and treatment preferences, whereas a minority reported significant changes over time [34]. One possible explanation for values shifts is values like ‘work’ or ‘pleasure’ might have become difficult to pursue or incompatible with common post-implantation goals: 1) cherishing time with loved ones, and 2) embracing a healthier lifestyle. Individuals reporting values stability might have already prioritized these values pre-implantation. This aligns with Schwartz’s theory of personal values, which categorizes 10 value types driven by different motivations (e.g., self-direction, hedonism), some of which conflict and impede attainment of one another [30, 31]. Essentially, individuals cannot pursue all their values simultaneously. Findings suggest clinicians should revisit patient values post-implantation to identify potential conflicts between prioritized values and LVAD-specific self-care activities.

Patients shared their values during broad, informal exchanges and focused, decision-making conversations to express their goals and priorities, convey their perspective, and justify specific choices to their caregivers and the healthcare team. Although many patients engaged in values discussions with their healthcare team, few recalled specific interactions or described them in much detail—initial LVAD implantation and consideration for heart transplantation were usually discussed. To our knowledge, studies in this population have focused exclusively on patient values during pre-implantation decision-making [35–39]. However, decision-making persists after implantation, requiring patients and families to navigate daily self-care and emergencies as they arise. Our findings highlight a need to characterize patient-clinician values discussions in the post-LVAD implantation period, including facilitators and barriers.

Patient values functioned as guiding and empowering forces for navigating life post-LVAD implantation. Patients often prioritized decisions aligned with ‘health’ and ‘family’ values over competing values like ‘pleasure’ or ‘work’. Inability to pursue one’s values emerged as a source of frustration. Similar reactions (i.e., frustration, anger) have been reported among patients with heart failure unable to pursue their values [40]. Our findings suggest patient values are instrumental to health decisions and coping following LVAD implantation. The results support the need for all clinicians—palliative care specialists and non-specialists—to effectively elicit and incorporate values into care [8, 41, 42]. Specifically, clinicians may facilitate identification of safe activities that meet as many of their prioritized values as possible.

### Strengths and limitations of the study

A strength of this study is that it is to our knowledge the first to specifically focus on patient values post-LVAD implantation [43], offering valuable insight into the values patients might prioritize post-implantation and their potential impact on patient self-care and coping. Additionally, recruitment from our study site, which is one of few regional centers providing LVAD support, facilitated inclusion of individuals with diverse backgrounds, including Black/African American individuals who are typically underrepresented in the LVAD literature. Finally, trustworthiness and authenticity were ensured through various strategies, including among others, maintaining an audit trail, providing thick descriptions, and performing member checks on final themes.

This study had several limitations. First, it was conducted at a single clinic. Future studies would be strengthened by participants across a range of sites and geographic regions, including those serving patients from other historically understudied LVAD populations (e.g., Hispanic/Latino, Asian). Second, patients were interviewed any point post-implantation, and values might continue to shift between early and late post-implantation. However, this is the first known study to consider patient values post-LVAD implantation [43]. Future research should examine values longitudinally from pre- to post-LVAD through end-of-life. Third, an open-ended values elicitation approach might have omitted relevant values since patients have been found to exclude values perceived as irrelevant to their healthcare (e.g., faith/religiosity) [16, 44]. Future work should consider a closed-ended values elicitation activity with the opportunity to add values. Finally, use of telephone interviews may have limited the richness of information produced by the interviews (e.g., conversation turns, word-density, field notes) [45]. However, telephone interviews are particularly useful in certain contexts, including unusual time demands or geographic distribution, which is applicable to our study sample who rely on their primary caregiver for transportation and many of whom live in neighboring states to the clinic [45].

### Implications and recommendations

Based on our study findings, we have several recommendations. First, clinicians inquire how a mentioned value (e.g., family) is impacting the patient’s self-care (e.g., diet, exercise) or how it might impact a decision under consideration (e.g., heart transplant). This may assist in assessing whether patient values conflict with LVAD-specific self-care tasks and facilitate shared understanding of patient goals/priorities when specific decisions must be made. Should conflicts arise, partner with the patient/family to identify an appropriate resolution. Second, clinicians assess for changes in family roles and coping post-implantation, noting any forgone activities. Clarification should occur if the patient can safely participate in an activity. If unsafe, partner with the patient/family to identify alternate activities. Third, research is conducted to identify desirable characteristics of values discussions from the patient perspective: preferred timing (before/during clinic visits), who they prefer to elicit their values, what topics they are comfortable discussing, and how they prefer to engage in values discussions (in-person, portal messages). Fourth, research is conducted to identify desirable characteristics of values discussions from the clinician perspective, given time constraints.

## Conclusion

LVAD implantation emerged as an impactful experience which prompted patients to reflect upon life and their values and priorities, including important relationships and their health/well-being. Patient values arose within broad, informal exchanges and focused, decision-making conversations with their caregiver and the healthcare team, and served as guiding and empowering forces for navigating life post-LVAD implantation. Inability to pursue one’s values emerged as a source of frustration. Given the influence patient values exert on health decisions and coping following LVAD implantation, patients must be supported, in part by recognizing certain values may shift or patients may be unable to pursue important activities/hobbies. Clinicians should assess the values of all patients post-implantation to facilitate shared understanding of their goals/priorities and identify potential changes in their coping.

### Supplementary Information

Additional file 1. Patient interview guide. The questions and prompts used to explore how LVAD recipients discuss, reflect upon, and act on their values.

Additional file 2. Sample page of audit trail entries. An example of audit trail entries to document research decisions.

## Data Availability

De-identified data are available from the corresponding author on reasonable request.

## Abbreviation

LVAD: Left ventricular assist device
