# Supplementary material for: “When I do have some time, rather than spend it polishing silver, I want to spend it with my grandkids”: a qualitative exploration of patient values following left ventricular assist device implantation
Source: BMC Palliat Care. 2024 May 22;23:128. doi: 10.1186/s12904-024-01454-y (PMC11110360; doi:10.1186/s12904-024-01454-y)
Supplement: Supplementary file 1 — Additional file 1. Patient interview guide. The questions and prompts used to explore how LVAD recipients discuss, reflect upon, and act on their values. [file 12904_2024_1454_MOESM1_ESM.docx]

Additional file 1. Patient Interview Guide

**PATIENT INTERVIEW GUIDE**

Revised 4/9/2023

**Stage 1 - Individual Interview Guide**

**Directions:** Sections written in *italics* are to be read to participants.

**Materials and Equipment**

2 digital recorders (need 2 recorders to prevent loss of data)

Extra batteries

1 pencil

1 pen

2 patient consent forms (1 for the patient, 1 for PI’s records)

**Time of Interview:**

**Date:**

**Place:**

**Interviewer:**

**Interviewee:**

**Introduction and Informed Consent**

Thank you for agreeing to speak with me today. The purpose of this study is to explore how people with left ventricular assist devices (LVADs) and their care partners talk about their values. Specifically, we are looking at how talking about values might affect how people cope with their condition and make health care decisions. As a person with an LVAD you have been selected as a participant for this study. Before we go through the study consent form, I hope to have 30 to 60 minutes of your time…is that what you expect? What time do you need to finish by? Let’s take a moment to go over the consent form for this study.

*Give the participant a copy of the consent form (or let them know they will receive one in the next few days if conducting a telephone interview). Ask them to follow along/listen as you read the form out loud. After reading the form, emphasize the following points:*

You have been invited to participate in a one-on-one interview about how individuals with a left ventricular assist device talk about their values. I invite you to share your personal views and experiences as they will help us better understand how talking about values may affect how individuals with an LVAD cope with their condition and make health care decisions. Your views and experiences will also provide us with important information that can be used to develop and test studies that can help patients to better match health decisions with a person’s values. Your participation in this study is voluntary and you may withdraw at any point during the session. What you say during this interview will not be shared with anyone outside of the research team.

I will be recording the interview because I don’t want to miss any of your comments or take any of your comments out of context. The recordings will be kept in a password protected database on the secure UAB School of Nursing server. We will remove your name or other identifying information mentioned during the interview. Your name will not be used in any reports of the findings. Do you have any questions for me before we start the interview and the recorder is turned on?

*Once all questions have been answered to their satisfaction, ask the participant to sign one copy of the consent form (or obtain verbal consent if conducting a telephone interview). If in-person, the participant should return the signed form to the interviewer. Each participant should keep the second copy of the consent form for their personal records. If by telephone, keep the verbally signed and dated copy of the consent and make a note to mail the participant a copy of the consent form.*

*After ensuring the door is closed, describe what is expected of the participant in terms of the interview process.*

Before I get into our discussion, please say exactly what’s on your mind. Everyone’s experiences are different. There are no right or wrong responses. Alright, let’s get started.

[TURN RECORDER ON]

**Introduction of Interview Questions/Topics** (total time: approximately 30-60 minutes)

*The interviewer will introduce each question, and explore it thoroughly before moving on to another question/topic. The interviewer will elicit opinions from the participant, and use probes as needed to assist the participant in providing detailed descriptions of their experiences and giving examples whenever possible. Questions are to be addressed in the following order:*

General Probes:

- Tell me more about…
- What do you mean by…?
- How did that make you feel?
- What were you thinking at the time/in that moment?
- Could you give an example?
- Please elaborate.

**General introductory questions:**

**Daily Management of LVAD**

1) To begin, I’d like to hear a little bit about your experience having an LVAD. What has this been like for you?

Probes:

- What does a typical day look like taking care of your LVAD?
- How has your daily routine changed since receiving an LVAD?
- What is the hardest part about living with an LVAD?
- What has been most surprising to you about living with an LVAD?

**Questions related to specific aims and research questions:**

**Reflection Upon Values**

Thank you. Next, I would like to ask you a few questions about your values. We know that individuals dealing with serious health issues sometimes reflect on their values as a way to help them deal with day to day stressors and decide how they want to live.

2) What does the word ‘values’ mean to you?

Probes:

- What things matter most to you?
- What things give your life meaning?

3) Since receiving your LVAD, what values have you thought about the most?

Probes:

- What values have been particularly meaningful to you since receiving your LVAD?
- What things have mattered most to you since you received your LVAD?

4) Since receiving your LVAD, how have your values changed or been re-prioritized, if at all?

Probes:

- What things have mattered most to you since you received your LVAD?
- [IF VALUES HAVE CHANGED] What specific experiences or life events do you think caused you to have different thoughts about your values?

**Values Discussions**

Now that I know a bit about your values, my next set of questions are going to focus on any conversations you may have had about your values with other people.

5) What has been your experience talking to **[care partner]** about your values?

[Yes, talked…] Probes:

- Tell me about those conversations.
- How did the conversation relate to any specific health-related or other life decisions?
- Who initiated the conversation? You or [**care partner**]?
- What changed, if anything, about your relationship with **[care partner]** after having those conversation?
- What was it like for you to have those conversations?

[No, have not talked…] Probes:

- What things got in the way from you and **[care partner]** having conversations about your values?
- What would it take for you to have this conversation with **[care partner]** in the future?

6) What has been your experience talking to your healthcare team about your values?

[Yes, talked…] Probes:

- Who was present during those conversations?
- What impact did the conversation have on a specific decision or treatment?
- Who initiated the conversation? You or [**care partner**] or the healthcare team?
- Who brought up your values during the conversation?
- What changed, if anything, about your relationship with the healthcare team after having those conversations?
- What was it like for you to have those conversations?

[No, have not talked…] Probes:

- What things got in the way from you and the healthcare team having conversations about your values?
- What would it take for you to have this conversation with the healthcare team in the future?

**Acting on Values**

The last set of questions have to do with how your values have affected any decisions and life changes since receiving an LVAD.

7) Since you had your LVAD implanted, what **decisions** have you had to make related to your health? These can be big or small decisions.

Probes:

- Earlier, you mentioned **[VALUE]** was important to you. How has **[VALUE]** affected how you make decisions related to your LVAD?
- How have **[care partner]**’s values affected how you make decisions related to your LVAD?

**Post LVAD Implantation Coping**

8) How do you usually deal with frustrating or stressful situations?

Probes:

- Earlier, you mentioned **[VALUE]** was important to you. How has **[VALUE]** affected how you deal with day to day life stressors?
- What makes it easier or harder for you to deal with everyday challenges?
- How well supported do you feel in dealing with frustrating or stressful situations?

9) [SKIP IF ANSWERED NO TO QUESTION 5] How has talking about your **values** with **[care partner]** affected how you deal with frustrating or stressful situations?

10) [SKIP IF ANSWERED NO TO QUESTION 6] How has talking about your **values** with your healthcare team affected how you deal with frustrating or stressful situations?

**Closing**

11) Are there any closing thoughts that you would like to share related to values conversations you have had with **[care partner]** or your healthcare team?

Okay, thank you so much for your time. I am going to stop the recorder now.

[STOP RECORDER].

How was the interview for you?

Okay to contact for follow up (Y/N)?

- Yes
- No

Preferred contact method

- Phone: ___________
- Email: ___________

Preferred time of day

- Morning
- Afternoon
- Evening

*Thank the participants for their time and participation in the interview. Reiterate their contribution to the study’s overall objective of improving care and support for patients with LVADs and their family caregivers.*

*Ask them if it is okay to contact them again for follow-up questions or to receive their input after data analysis. Let them know that the PI’s contact information is found on their consent form.*

**INTERVIEWER REFLECTION**

**(indicate interviewee ID# and add to digital recording)**

*After the interview, record responses to the following questions in private.*

| 1. In general, HOW DID THE INTERVIEW GO? (e.g. how engaged was the participant, how candid were their responses, how was my own comfort level) |
| --- |
| 1. What MAIN TOPICS came up? (brief points) |
| 1. What NEW information came up? |
| 1. What information was SURPRISING? |
| 1. What things were CONFIRMED or CHALLENGED in this interview? |
| 1. Should anything about the interview guide or process be CHANGED? |
