# Supplementary material for: “When I do have some time, rather than spend it polishing silver, I want to spend it with my grandkids”: a qualitative exploration of patient values following left ventricular assist device implantation
Source: BMC Palliat Care. 2024 May 22;23:128. doi: 10.1186/s12904-024-01454-y (PMC11110360; doi:10.1186/s12904-024-01454-y)
Supplement: Supplementary file 2 — Additional file 2. Sample page of audit trail entries. An example of audit trail entries to document research decisions. [file 12904_2024_1454_MOESM2_ESM.docx]

Additional file 2. Sample Page of Audit Trail Entries

**Audit Trail**

***Categories***

- Personal: How my unique perspectives are influencing the research
- Interpersonal: What relationships exist, how they are influencing the research/people involved, power dynamics at play
- Methodological: How I am making methodological decisions, their implications
- Contextual: How aspects of context are influencing the research and people involved
- Data Analysis: Reflections about data analysis
- Final Report: Considerations pertaining to the final report of the study
- Future Directions: Potential future directions for this work

| **Date/Time** | **Memos/Entries** |
| --- | --- |
| 1/26/2023 1:51pm | For today’s note, I think it is most helpful to reflect on my own experiences and expectations. My clinical background is as a care assistant in nursing homes and a nurse in acute care settings. I have approximately 1.5 years of clinical experience working with patients with heart failure. […] I have never provided care for patients with an LVAD. However, I have cared for many patients pre- and post-CABG and with pacemakers and ICDs. I am familiar with heart failure patients facing (or who have previously faced) complex medical decisions and with cardiac devices. I realize my lack of direct patient care makes me a bit uncomfortable doing research with the LVAD population. Although, the purpose of this study is to explore the experiences of VAD patients and caregivers, so not having too many assumptions/expectations could be a good thing. This is something I spoke with Dr. JNO about and he said it is not unusual for researchers to conduct research in a population they did not provide direct care for—also, I suppose that is the case for non-clinician researchers. I should try not to see this as a weakness but a learning opportunity and area of growth. Dr. JNO suggested that I consider some learning opportunities to supplement my knowledge in addition to readings. [Personal] |
| 2/23/2023 3:00pm | The VAD coordinators have been introducing me to the patient (and any family present) and indicating that I will be telling them about a research study. While there doesn’t seem to be too much influence being exerted on the participants, I should be wary of this while analyzing the values discussions section, particularly regarding patient-clinician and caregiver-clinician discussions. One of the survey questions asks about their reason for participation, so I might be able to capture any relationships influencing the study in this way. [Interpersonal] |
| 6/22/2023 3:45pm | ***PT 16***  I’ve been thinking about why some participants say everything seemed more important since the LVAD was implanted. After some digging, what I found sounds a lot like near-death experiences (Life After Death: A Narrative Exploration of Near-Death Experiences), which can generate “*shifts in awareness, priorities, and relationships*” The findings from a narrative study, particularly the following passage resonates with my findings, and makes me think LVAD implantation may be perceived as a near-death experience: “*Following a near-death experience, participants discussed re-evaluation of life goals and meaning.* *This realization inspired a new lens through which participants saw life and how they approached their goals, relationships, and everyday lives. Extrinsic goals such as material possessions became less important than intrinsic needs. Participants sought meaningful relationships over superficial connections*.” Several participants mentioned being very sick and implanted emergently, so I think this is a possibility. PT16 also vividly described what sounded like a near-death experience. [Data analysis] |
| 10/3/2023 4:21pm | In reviewing the patient themes, Dr. JNO had some suggestions. For the Basic Theme ‘Values are conveyed to help rationalize a particular decision option’, he asked “Conveyed to who?”. This is a good point of clarification. I have revised the theme to ‘Values are conveyed to the care partner help rationalize a particular decision option’. [Data analysis] |
